# Supplementary material for: Circulating Peptidome Is Strongly Altered in COVID-19 Patients
Source: Int J Environ Res Public Health. 2023 Jan 14;20(2):1564. doi: 10.3390/ijerph20021564 (PMC9865723; doi:10.3390/ijerph20021564)
Supplement: Supplementary file 1 [file ijerph-20-01564-s001.zip › ijerph-2071854-supplementary-Tables S1 and S2 and Figures S1-S13.pdf]

Supplementary Materials

Table S1. Complete list of all modulated peptides in COVID-19 patients vs negative controls.

Peptide Sequence	Protein	Modulation	*p*-Value	Fold Change
HKSEVAHRFKDLGEENFKALVL	ALBU_HUMAN	COVID-19/Negative	1.4819×10^{-7}	0.03603455
ESFKVSFLSALEEYTKKLNTQ	APOA1_HUMAN	COVID-19/Negative	8.9797×10^{-6}	0.072533931
SPMYSIITPNILRLESEETM	CO3_HUMAN	COVID-19/Negative	0.000023028	0.064445619
HKSEVAHRFKDLGEENFKALVLIA	ALBU_HUMAN	COVID-19/Negative	0.000058963	0.05
LLSPYSYSTTAVVTNPKE	TTHY_HUMAN	COVID-19/Negative	0.00011	0.177978517
LSALEEYTKKLNTQ	APOA1_HUMAN	COVID-19/Negative	0.00015	0.119789623
SGFLLFPDMEA	C1QB_HUMAN	COVID-19/Negative	0.00038	0.05
FKVSFLSALEEYTKKLNTQ	APOA1_HUMAN	COVID-19/Negative	0.0005	0.093010926
MIEQNTKSPLFMGKVVNPTQK	A1AT_HUMAN	COVID-19/Negative	0.00051	0.167694733
FEIPINGLSEF	ITIH2_HUMAN	COVID-19/Negative	0.00065	0.05
AHKSEVAHRFKDLGEENFKALVL	ALBU_HUMAN	COVID-19/Negative	0.00072	0.05
FEIPINGLSE	ITIH2_HUMAN	COVID-19/Negative	0.001	0.024817714
LEEYTKKLNTQ	APOA1_HUMAN	COVID-19/Negative	0.001	0.05
SEVAHRFKDLGEENFKALVL	ALBU_HUMAN	COVID-19/Negative	0.00114	0.05
SPMYSIITPNILRLESEETMVL	CO3_HUMAN	COVID-19/Negative	0.00143	0.05
DAHKSEVAHRFKDLGEENFKALVLIA	ALBU_HUMAN	COVID-19/Negative	0.00162	0.197839412
FVEAVEQNTLQEFLKLA	SH3L3_HUMAN	COVID-19/Negative	0.00173	0.146787861
TANDSGPRRYTIAALLSPYSYSTTAVVTNPKE	TTHY_HUMAN	COVID-19/Negative	0.00174	0.080120812
SEAEDASLLSFMQG	APOC3_HUMAN	COVID-19/Negative	0.00199	0.134397761
LGEENFKALVL	ALBU_HUMAN	COVID-19/Negative	0.00281	0.091110812
DAHKSEVAHRFKDLGEENFKALVLIAF	ALBU_HUMAN	COVID-19/Negative	0.00351	0.016668508
FQVLPWLKEKLQDEDLGFL	CFAB_HUMAN	COVID-19/Negative	0.00423	0.05
AKLIALTL	PON1_HUMAN	COVID-19/Negative	0.00467	0.05
YSMRKMSMKIRPFFPQQ	FIBB_HUMAN	COVID-19/Negative	0.00504	0.05
DLGEENFKALVL	ALBU_HUMAN	COVID-19/Negative	0.00664	0.05
DDPDAPLQPVTPLQL	CO4A_HUMAN	COVID-19/Negative	0.00665	0.336561131
DAHKSEVAHRFKDLGEENFKAL	ALBU_HUMAN	COVID-19/Negative	0.00676	0.113495694
YVKVTSIQDWVQKTIAEN	HPT_HUMAN I	COVID-19/Negative	0.00748	0.210010953
FKVSFLSALEE	APOA1_HUMAN	COVID-19/Negative	0.00752	0.055194246
PDAPLQPVTPLQL	CO4A_HUMAN	COVID-19/Negative	0.0076	0.053651856
ALLSPYSYSTTAVVTNPKE	TTHY_HUMAN	COVID-19/Negative	0.00769	0.327026104
LEAIPMSIPPEVKFNKPFVF	A1AT_HUMAN	COVID-19/Negative	0.0092	0.126063994
LMIEQNTKSPLFMGKVVNPTQK	A1AT_HUMAN	COVID-19/Negative	0.00922	0.252785733
DAHKSEVAHRFKDLGEENFKALVL	ALBU_HUMAN	COVID-19/Negative	0.00934	0.393735358
IEQNTKSPLFMGKVVNPTQK	A1AT_HUMAN	COVID-19/Negative	0.00978	0.226261841
DAHKSEVAHRFKDLGEENFKA	ALBU_HUMAN	COVID-19/Negative	0.01002	0.05
FKVSFLSALEEYTKKLNT	APOA1_HUMAN	COVID-19/Negative	0.01052	0.060287794
VHLTPEEKSAVTALWGKVNVDEVGGEALGRL	HBB_HUMAN H	COVID-19/Negative	0.01067	0.05

SEAEDASLLSFMQGYMKHATKTAKDALSSVQESQVAQQARGWVTDGFSSLKDYWSTVKDKFSEF	APOC3_HUMAN	COVID-19/Negative	0.01103	0.049693447
GEENFKALVLIA	ALBU_HUMAN	COVID-19/Negative	0.01104	0.05
VTLAAHLPAEFTPAVHASLDKFLASVSTVLTSKYR	HBA_HUMAN H	COVID-19/Negative	0.01114	0.05
GEENFKALVL	ALBU_HUMAN	COVID-19/Negative	0.01141	0.068964642
SLMPFSPYEPLNF	CLUS_HUMAN	COVID-19/Negative	0.01228	0.171279459
IQSKVVNNSPQPQNVVF	ITIH2_HUMAN	COVID-19/Negative	0.01238	0.097022154
VDSGNDVTDIADD	HPT_HUMAN I	COVID-19/Negative	0.01483	50.72550517
SPMYSIITPNILRLESEE	CO3_HUMAN C	COVID-19/Negative	0.01524	0.257139328
STTAVVTNPKE	TTHY_HUMAN	COVID-19/Negative	0.01529	5.412501503
SEAEDASL	APOC3_HUMAN	COVID-19/Negative	0.01647	8.234697164
WDLDPEVRPTSAVAA	APOC3_HUMAN	COVID-19/Negative	0.01722	0.175998849
PVLESFKVSFLSALEEYTKKLNTQ	APOA1_HUMAN	COVID-19/Negative	0.01817	0.119474519
VELAPGKFQLVAENRRYQ	ITIH2_HUMAN	COVID-19/Negative	0.01889	0.05
SPMYSIITPNILRLESEET	CO3_HUMAN C	COVID-19/Negative	0.01977	0.192688842
DEPPQSPWDRVKDLATVYVDVLKDSGRD	APOA1_HUMAN	COVID-19/Negative	0.02078	0.05
LSALEEYTKKLNT	APOA1_HUMAN	COVID-19/Negative	0.02082	0.05
TIDEKGTEAAGAMFL	A1AT_HUMAN	COVID-19/Negative	0.0227	0.05
YVVKVFSLAVNL	CO3_HUMAN	COVID-19/Negative	0.0228	0.05
TVLTSKYR	HBA_HUMAN	COVID-19/Negative	0.02763	0.05
MMEEVDQVTL	ITIH2_HUMAN	COVID-19/Negative	0.03031	0.05
VAFDLEIPKTAFISD	ITIH1_HUMAN	COVID-19/Negative	0.03204	0.05
DAHKSEVAHRFKDLGEENFKAWAV	ALBU_HUMAN	COVID-19/Negative	0.03277	0.171071235
DAHKSEVAHRFKDLGEENFKALVLIAFAQY	ALBU_HUMAN	COVID-19/Negative	0.03332	0.05
DIVMTQTPLSL	KVD29_HUMAN	COVID-19/Negative	0.03442	0.05
SALEEYTKKLNT	APOA1_HUMAN	COVID-19/Negative	0.04125	0.125538404
SLPLLMDSVIQAL	PGRP2_HUMAN	COVID-19/Negative	0.04227	0.05
DSGEGDFLAEGGGVR	FIBA_HUMAN	COVID-19/Negative	0.0431	6.508456648
KPRLLLFSPSVVHLGVPLSVGVQLQDVPRGQVVKGSVF	CO4A_HUMAN	COVID-19/Negative	0.04432	0.198083574
TLEIPGNSD	CO4A_HUMAN	COVID-19/Negative	0.04436	20
YGVYVKVTSIQDWVQKTIAEN	HPT_HUMAN	COVID-19/Negative	0.04538	0.05
TVLQNEDTKSG	CERU_HUMAN	COVID-19/Negative	0.0463	5.683018242
ANPGLVARITDKGLQYAAQEGLLALQSEL	LBP_HUMAN	COVID-19/Negative	0.04721	0.05
EIVLTQSPATL	KVD11_HUMAN	COVID-19/Negative	0.04895	0.216524448
VMLLDTWDQVF	GELS_HUMAN	COVID-19/Negative	0.04922	0.05
SPMYSIITPNIL	CO3_HUMAN	COVID-19/Negative	0.04931	0.272890958

Table S2. Complete list of all modulated peptides in mild COVID-19 patients vs negative controls and in severe COVID-19 patients vs negative controls.

Peptide Sequence	Protein	Modulation	*p*-Value	Fold Change
HKSEVAHRFKDLGEENFKALVL	ALBU_HUMAN	Mild/Negative	4.33×10^{-5}	0.05
LLSPYSYSTTAVVTNPKE	TTHY_HUMAN	Mild/Negative	7.96×10^{-5}	0.01473138
LSALEEYTKKLNTQ	APOA1_HUMAN	Mild/Negative	0.00032	0.05
SPMYSIITPNILRLESEE	CO3_HUMAN	Mild/Negative	0.00032	0.05
SPMYSIITPNILRLESEETM	CO3_HUMAN	Mild/Negative	0.00034	0.05
ESFKVSFLSALEEYTKKLNTQ	APOA1_HUMAN	Mild/Negative	0.00053	0.05416258
DAHKSEVAHRFKDLGEENFKALVL	ALBU_HUMAN	Mild/Negative	0.00221	0.19192929
FVEAVEQNTLQEFLKLA	SH3L3_HUMAN	Mild/Negative	0.00253	0.05
VDSGNDVTDIADD	HPT_HUMAN	Mild/Negative	0.00254	18.7629557
SEAEDASL	APOC3_HUMAN	Mild/Negative	0.00321	6.53240179
HKSEVAHRFKDLGEENFKALVLIA	ALBU_HUMAN	Mild/Negative	0.00329	0.05
IEQNTKSPLFMGKVVNPTQK	A1AT_HUMAN	Mild/Negative	0.00342	0.05
SEAEDASLLSFMQG	APOC3_HUMAN	Mild/Negative	0.00447	0.05
FKVSFLSALEEYTKKLNTQ	APOA1_HUMAN	Mild/Negative	0.00496	0.03713498
DEAGSEADHEGTHST	FIBA_HUMAN	Mild/Negative	0.00546	3.86064875
ALLSPYSYSTTAVVTNPKE	TTHY_HUMAN	Mild/Negative	0.00576	0.16790454
MIEQNTKSPLFMGKVVNPTQK	A1AT_HUMAN	Mild/Negative	0.00592	0.1204362
SPMYSIITPNIL	CO3_HUMAN	Mild/Negative	0.00804	0.05
TANDSGPRRYTIAALLSPYSYSTTAVVTNPKE	TTHY_HUMAN	Mild/Negative	0.00862	0.05
DAHKSEVAHRFKDLGEENFKALVLIA	ALBU_HUMAN	Mild/Negative	0.00965	0.12242114
SGFLLFPDMEA	C1QB_HUMAN	Mild/Negative	0.00968	0.05
EIVLTQSPATL	KVD11_HUMAN	Mild/Negative	0.00972	0.05
LGEENFKALVL	ALBU_HUMAN	Mild/Negative	0.01009	0.05
DSGEGDFLAEGGGV	FIBA_HUMAN	Mild/Negative	0.01195	4.11234182
FEIPINGLSEF	ITIH2_HUMAN	Mild/Negative	0.01328	0.05
VKVTSIQDWVQKTIAEN	HPT_HUMAN	Mild/Negative	0.01329	0.15143888
DAHKSEVAHRFKDLGEENFKAL	ALBU_HUMAN	Mild/Negative	0.01376	0.05
AHKSEVAHRFKDLGEENFKALVL	ALBU_HUMAN	Mild/Negative	0.01406	0.05
FVELGTQPATQ	APOA2_HUMAN	Mild/Negative	0.01444	4.61784331
YVKVTSIQDWVQKTIAEN	HPT_HUMAN	Mild/Negative	0.01457	0.10936587
GVYVKVTSIQDWVQKTIAEN	HPT_HUMAN	Mild/Negative	0.01496	0.07093165
FDTASTGKTFPGFFSPMLGEF	FIBA_HUMAN	Mild/Negative	0.01576	0.12726136
LEEYTKKLNTQ	APOA1_HUMAN	Mild/Negative	0.01699	0.05
KPRLLLFSPSVVHLGVPLSVGVQLQDVPRGQVVKGSVF	CO4A_HUMAN	Mild/Negative	0.01762	0.05
SEVAHRFKDLGEENFKALVL	ALBU_HUMAN	Mild/Negative	0.01828	0.05
LMIEQNTKSPLFMGKVVNPTQK	A1AT_HUMAN	Mild/Negative	0.01926	0.1080784
FEIPINGLSE	ITIH2_HUMAN	Mild/Negative	0.01937	0.04737927
SPMYSIITPNILRLESEETMVL	CO3_HUMAN	Mild/Negative	0.02085	0.05

DAHKSEVAHRFKDLGEENFKAWAV	ALBU_HUMAN	Mild/Negative	0.02373	0.05
SEAEDASLLS	APOC3_HUMAN	Mild/Negative	0.02524	6.39603328
KPRLLLFSPSVVHLGVPLSVG	CO4A_HUMAN	Mild/Negative	0.02564	0.05
LEAIPMSIPPEVKFNKPFVF	A1AT_HUMAN	Mild/Negative	0.02725	0.05
FVLPKFEVQVTVPKIITIL	A2MG_HUMAN	Mild/Negative	0.02752	0.05
EAIPMSIPPEVKFNKPFVF	A1AT_HUMAN	Mild/Negative	0.03188	0.13070053
IQSKVVNNSPQPQNVVF	ITIH2_HUMAN	Mild/Negative	0.03392	0.05
PVLESFKVSFLSALEEYTKKLNTQ	APOA1_HUMAN	Mild/Negative	0.03428	0.05
DAHKSEVAHRFKDLGEENFKALVLIAF	ALBU_HUMAN	Mild/Negative	0.03752	0.0318217
FKVSFLSALEE	APOA1_HUMAN	Mild/Negative	0.03794	0.05
FQVLPWLKEKLQDEDLGFL	CFAB_HUMAN	Mild/Negative	0.03892	0.05
WDLDPEVRPTSAVAA	APOC3_HUMAN	Mild/Negative	0.04015	0.112474
LSPYSYSTTAVVTNPKE	TTHY_HUMAN	Mild/Negative	0.04049	0.27417333
AKLIALTL	PON1_HUMAN	Mild/Negative	0.04118	0.05
YSMRKMSMKIRPFFPQQ	FIBB_HUMAN	Mild/Negative	0.04302	0.05
FKVSFLSAL	APOA1_HUMAN	Mild/Negative	0.04415	0.05
GEENFKALVL	ALBU_HUMAN	Mild/Negative	0.04442	0.05
DSGEGDFLAEGGGVR	FIBA_HUMAN	Mild/Negative	0.04475	4.92842385
FKVSFLSALEEYTKKLNT	APOA1_HUMAN	Mild/Negative	0.04552	0.05
DTASTGKTFPGFFSPMLGEF	FIBA_HUMAN	Mild/Negative	0.04724	0.05
DEAGSEADHEGTHST	FIBA_HUMAN	Severe/Negative	9.03×10^{-5}	0.05
HKSEVAHRFKDLGEENFKALVL	ALBU_HUMAN	Severe/Negative	0.00027	0.07567255
DDPDAPLQPVTPLQ	CO4A_HUMAN	Severe/Negative	0.00078	0.05
STTAVVTNPKE	TTHY_HUMAN	Severe/Negative	0.00078	6.60664002
ESFKVSFLSALEEYTKKLNTQ	APOA1_HUMAN	Severe/Negative	0.00177	0.09274242
VDSGNDVTDIADD	HPT_HUMAN	Severe/Negative	0.00186	85.8843096
HKSEVAHRFKDLGEENFKALVLIA	ALBU_HUMAN	Severe/Negative	0.00499	0.05
SPMYSIITPNILRLESEETM	CO3_HUMAN	Severe/Negative	0.00516	0.1353358
WVQKTIAEN	HPT_HUMAN	Severe/Negative	0.00546	13.4420523
AVVTNPKE	TTHY_HUMAN	Severe/Negative	0.01157	4.38773378
TVLQNEDTKSG	CERU_HUMAN	Severe/Negative	0.01234	5.25024235
SGFLLFPDMEA	C1QB_HUMAN	Severe/Negative	0.01355	0.05
LSYFVELGTQPATQ	APOA2_HUMAN	Severe/Negative	0.01458	0.05
ALEILQEEDL	CO4A_HUMAN	Severe/Negative	0.01548	4.63441985
SEETKENEGFTVTAEG	CO3_HUMAN	Severe/Negative	0.01599	0.05
MIEQNTKSPLFMGKVVNPTQK	A1AT_HUMAN	Severe/Negative	0.01697	0.21967912
FEIPINGLSEF	ITIH2_HUMAN	Severe/Negative	0.01815	0.05
DDPDAPLQPVTPLQL	CO4A_HUMAN	Severe/Negative	0.01823	0.22859148
AHKSEVAHRFKDLGEENFKALVL	ALBU_HUMAN	Severe/Negative	0.01914	0.05
FEIPINGLSE	ITIH2_HUMAN	Severe/Negative	0.01933	0.05
SPMYSIITPNILRLESEET	CO3_HUMAN	Severe/Negative	0.01946	0.06530704
LSALEEYTKKLNTQ	APOA1_HUMAN	Severe/Negative	0.01997	0.25155821

SEETKENEGFTVTAEGK	CO3_HUMAN	Severe/Negative	0.02143	0.05
SGASGPENFQVG	TLN1_HUMAN	Severe/Negative	0.02202	20
FKVSFLSALEEYTKKLNTQ	APOA1_HUMAN	Severe/Negative	0.02213	0.15447447
LEEYTKKLNTQ	APOA1_HUMAN	Severe/Negative	0.0228	0.05
LLSPYSYSTTAVVTNPKE	TTHY_HUMAN	Severe/Negative	0.02388	0.35755037
SEVAHRFKDLGEENFKALVL	ALBU_HUMAN	Severe/Negative	0.0244	0.05
TLEIPGNSD	CO4A_HUMAN	Severe/Negative	0.02502	20
SEAEDASL	APOC3_HUMAN	Severe/Negative	0.02521	10.1072221
SPMYSIITPNILRLESEETMVL	CO3_HUMAN	Severe/Negative	0.02755	0.05
SLMPFSPYEPLNF	CLUS_HUMAN	Severe/Negative	0.02901	0.08849264
EDPQGDAAQKTDT	A1AT_HUMAN	Severe/Negative	0.03527	0.05
DSGEGDFLAEGGGVR	FIBA_HUMAN	Severe/Negative	0.03778	8.24649273
DAHKSEVAHRFKDLGEENFKALVLIAF	ALBU_HUMAN	Severe/Negative	0.04076	0.05
DAHKSEVAHRFKDLGEENFKALVLIA	ALBU_HUMAN	Severe/Negative	0.04448	0.28079951
TANDSGPRRYTIAALLSPYSYSTTAVVTNPKE	TTHY_HUMAN	Severe/Negative	0.04739	0.16825371
PDAPLQPVTPLQL	CO4A_HUMAN	Severe/Negative	0.04882	0.05
FQVLPWLKEKLQDEDLGFL	CFAB_HUMAN	Severe/Negative	0.04904	0.05

MS/MS Fragmentation of **MIEQNTKSPLFMGKVVNPTQK**
Found in **sp|P01009|A1AT_HUMAN** in **uniprot-humanREV_NEW_**, sp|P01009|A1AT_HUMAN Alpha-1-antitrypsin OS=Homo sapiens GN=SERPINA1 PE=1 SV=3

Match to Query 1227: 2389.244696 from(598.318450,4+) rtinseconds(731) index(412)
Title: Locus:1.1.1.526.2 File:"Peptidi Plasma COVID19_NEG41_LIB1.wiff"
Data file Peptidi Plasma COVID19_NEG41_LIB1.mgf

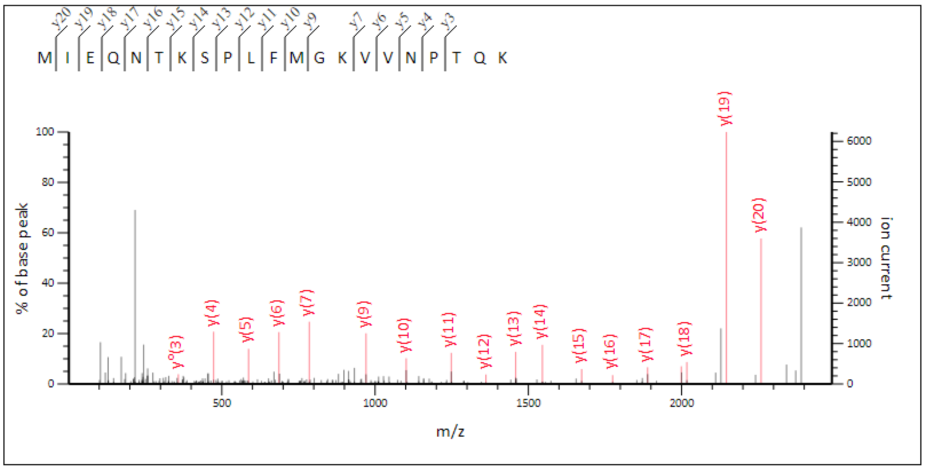

Figure S1. MS/MS fragmentation of the peptide MIEQNTKSPLFMGKVVNPTQK.

MS/MS Fragmentation of **SPMYSIITPNILRLESEETMVL**
Found in **sp|P01024|CO3_HUMAN** in **uniprot-humanREV_NEW_**, sp|P01024|CO3_HUMAN Complement C3 OS=Homo sapiens GN=C3 PE=1 SV=2

Match to Query 1284: 2535.291102 from(846.104310,3+) rtinseconds(1376) index(1191)
Title: Locus:1.1.1.696.4 File:"Peptidi Plasma COVID19_NEG41_LIB1.wiff"
Data file Peptidi Plasma COVID19_NEG41_LIB1.mgf

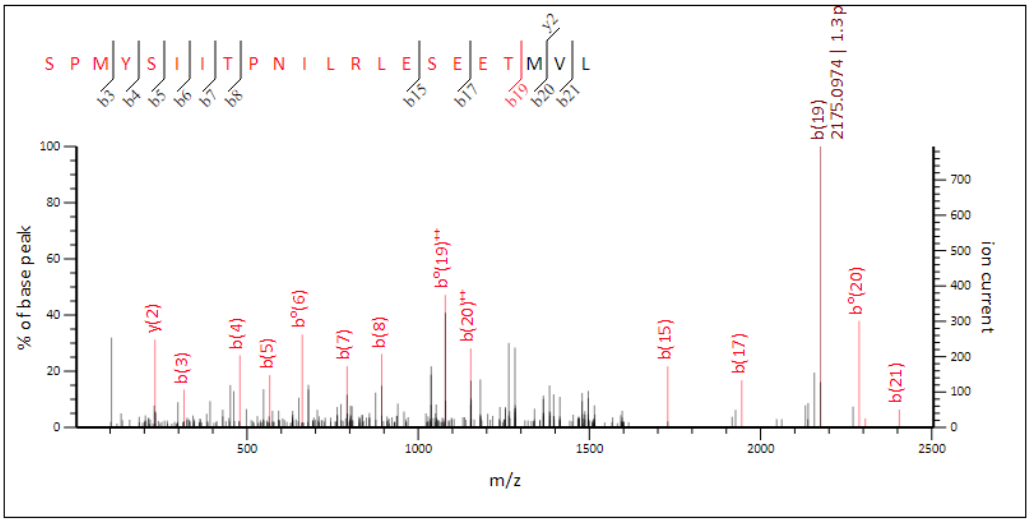

Figure S2. MS/MS fragmentation of the peptide SPMYSIITPNILRLESEETMVL.

MS/MS Fragmentation of **FEIPINGLSE**
Found in **sp|P19823|ITIH2_HUMAN** in **uniprot-humanREV_NEW_**, sp|P19823|ITIH2_HUMAN Inter-alpha-trypsin inhibitor heavy chain H2 OS=Homo sapiens GN=ITIH2 PE=1 SV=2

Match to Query 757: 1117.567288 from(559.790920,2+) rtinseconds(1073) index(903)
Title: Locus:1.1.1.614.7 File:"Peptidi Plasma COVID19_NEG41_LIB1.wiff"
Data file Peptidi Plasma COVID19_NEG41_LIB1.mgf

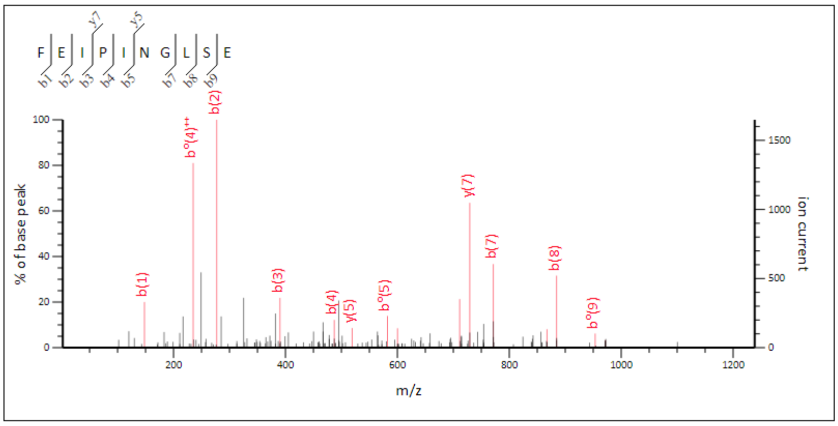

Figure S3. MS/MS fragmentation of the peptide FEIPINGLSE.

MS/MS Fragmentation of **SGFLLFPDMEA**
Found in **sp|P02746|C1QB_HUMAN** in **uniprot-humanREV_NEW_**, sp|P02746|C1QB_HUMAN Complement C1q subcomponent subunit B OS=Homo sapiens GN=C1QB PE=1 SV=3

Match to Query 830: 1225.565828 from(613.790190,2+) rtinseconds(1362) index(1181)
Title: Locus:1.1.1.692.3 File:"Peptidi Plasma COVID19_NEG41_LIB1.wiff"
Data file Peptidi Plasma COVID19_NEG41_LIB1.mgf

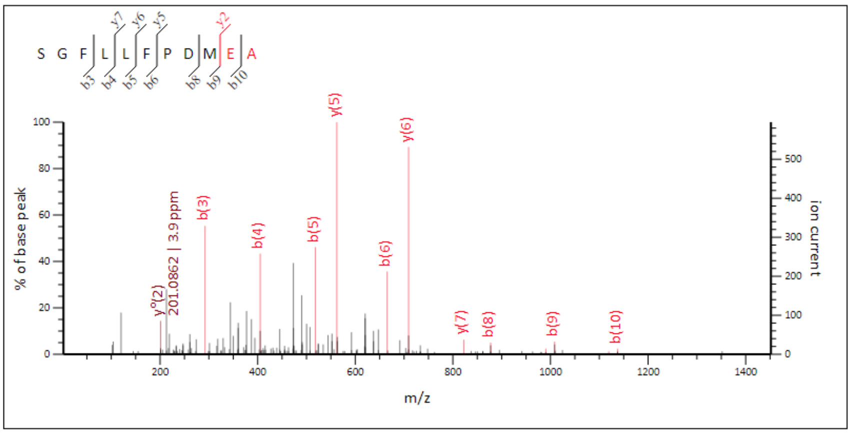

Figure S4. MS/MS fragmentation of the peptide SGFLLFPDMEA.

MS/MS Fragmentation of **TVLQNEDTKSG**
Found in **sp|P00450|CERU_HUMAN** in **uniprot-humanREV_NEW_**, sp|P00450|CERU_HUMAN Ceruloplasmin OS=Homo sapiens GN=CP PE=1 SV=1

Match to Query 515: 1190.580728 from(596.297640,2+) rtinseconds(303) index(138)
Title: Locus:1.1.1.338.4 File:"Peptidi Plasma COVID19_MILD_CE_07072021_LIB1.wiff"
Data file Peptidi Plasma COVID19_MILD_CE_07072021_LIB1.mgf

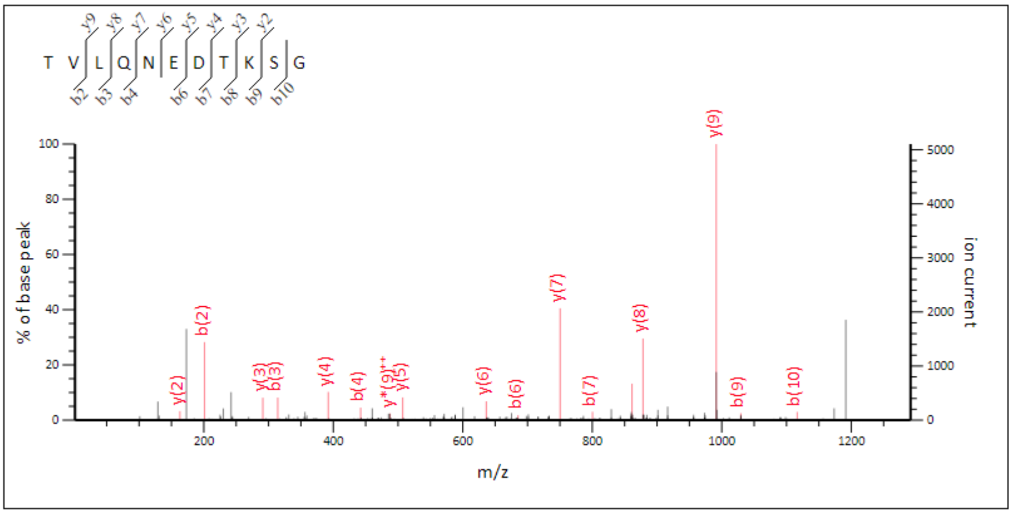

Figure S5. MS/MS fragmentation of the peptide TVLQNEDTKSG.

MS/MS Fragmentation of **VDSGNDVTDIADD**
Found in **sp|P00738-2|HPT_HUMAN** in **uniprot-humanREV_NEW_**, sp|P00738-2|HPT_HUMAN Isoform 2 of Haptoglobin OS=Homo sapiens GN=HP

Match to Query 559: 1334.548608 from(668.281580,2+) rtinseconds(614) index(315)
Title: Locus:1.1.1.483.4 File:"Peptidi Plasma COVID19_MILD_CE_07072021_LIB1.wiff"
Data file Peptidi Plasma COVID19_MILD_CE_07072021_LIB1.mgf

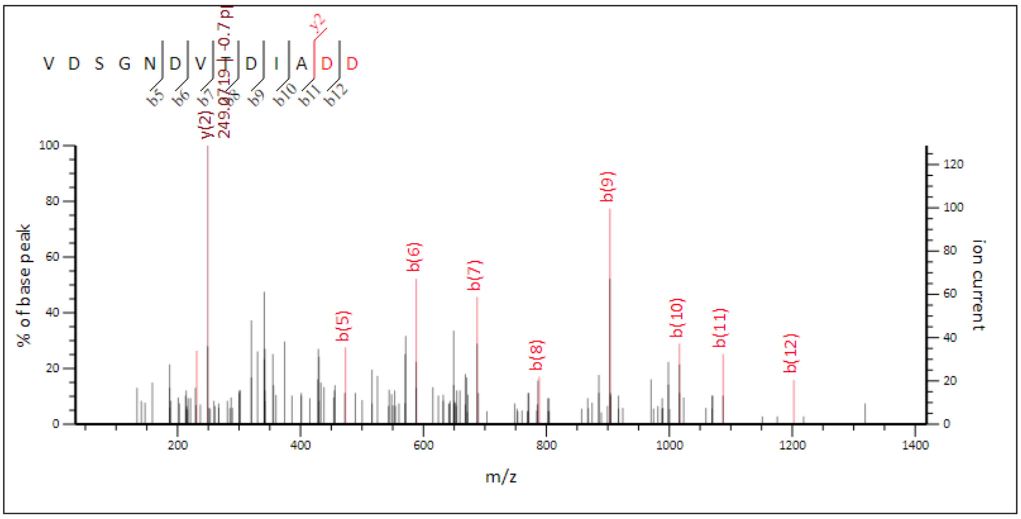

Figure S6. MS/MS fragmentation of the peptide VDSGNDVTDIADD.

MS/MS Fragmentation of **STTAVVTNPKE**
Found in **sp|P02766|TTHY_HUMAN** in **uniprot-humanREV_NEW_**, sp|P02766|TTHY_HUMAN Transthyretin OS=Homo sapiens GN=TTR PE=1 SV=1

Match to Query 495: 1145.595148 from(573.804850,2+) rtinseconds(359) index(171)
Title: Locus:1.1.1.363.2 File:"Peptidi Plasma COVID19_MILD_CE_07072021_LIB1.wiff"
Data file Peptidi Plasma COVID19_MILD_CE_07072021_LIB1.mgf

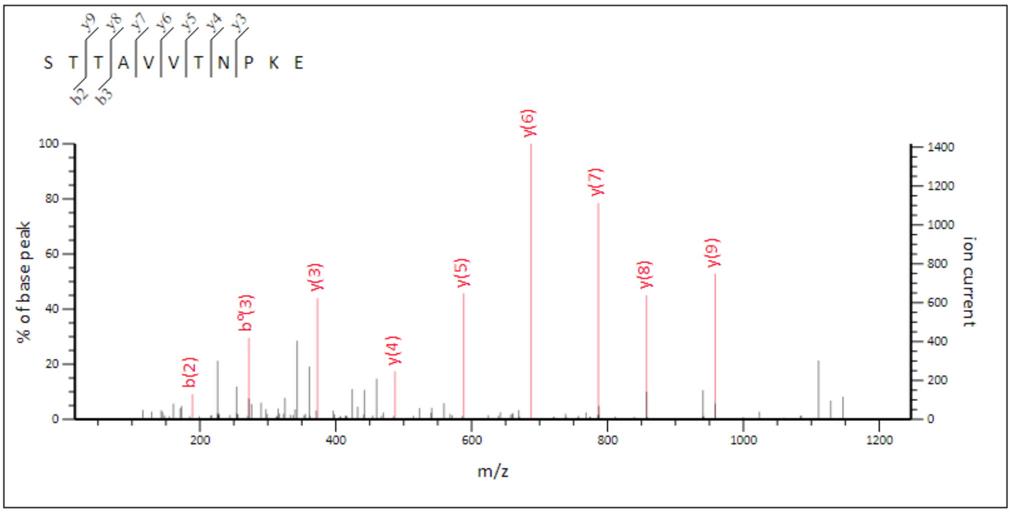

Figure S7. MS/MS fragmentation of the peptide STTAVVTNPKE.

MS/MS Fragmentation of **DSGEGDFLAEGGGVR**
Found in **sp|P02671-2|FIBA_HUMAN** in **uniprot-humanREV_NEW_**, sp|P02671-2|FIBA_HUMAN Isoform 2 of Fibrinogen alpha chain OS=Homo sapiens GN=FGA

Match to Query 581: 1464.650188 from(733.332370,2+) rtinseconds(691) index(361)
Title: Locus:1.1.1.513.5 File:"Peptidi Plasma COVID19_MILD_CE_07072021_LIB1.wiff"
Data file Peptidi Plasma COVID19_MILD_CE_07072021_LIB1.mgf

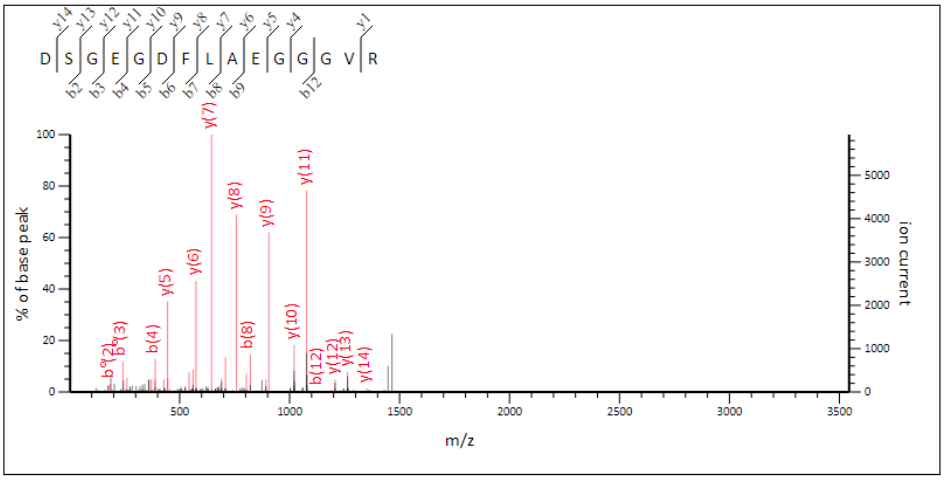

Figure S8. MS/MS fragmentation of the peptide DSGEGDFLAEGGGVR.

MS/MS Fragmentation of **TLEIPGNSD**
Found in **sp|P0C0L5|CO4B_HUMAN** in **uniprot-humanREV_NEW_**, sp|P0C0L5|CO4B_HUMAN Complement C4-B OS=Homo sapiens GN=C4B PE=1 SV=2

Match to Query 256: 944.442088 from(473.228320,2+) rtinseconds(652) index(205)
Title: Locus:1.1.1.630.2 File:"Peptidi Plasma COVID19_SEVERE_15_08072021_LIB1.wiff"
Data file Peptidi Plasma COVID19_SEVERE_15_08072021_LIB1.mgf

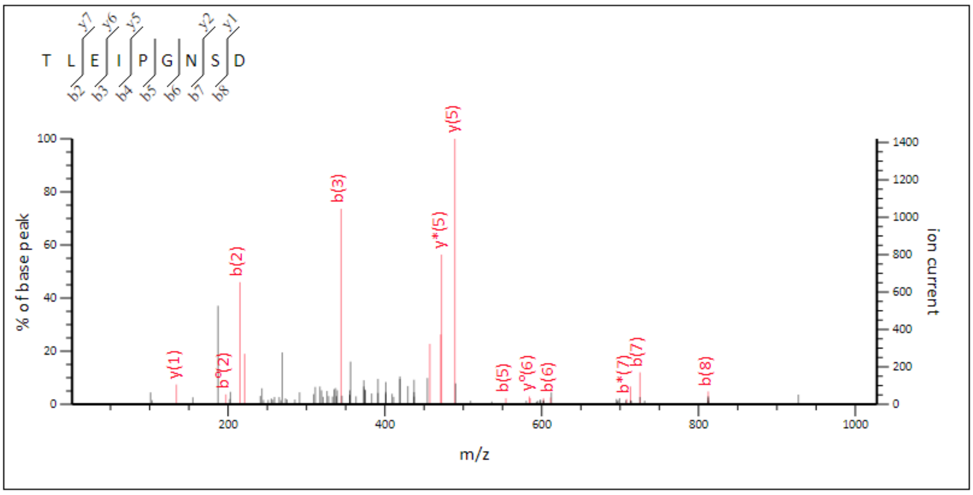

Figure S9. MS/MS fragmentation of the peptide TLEIPGNSD.

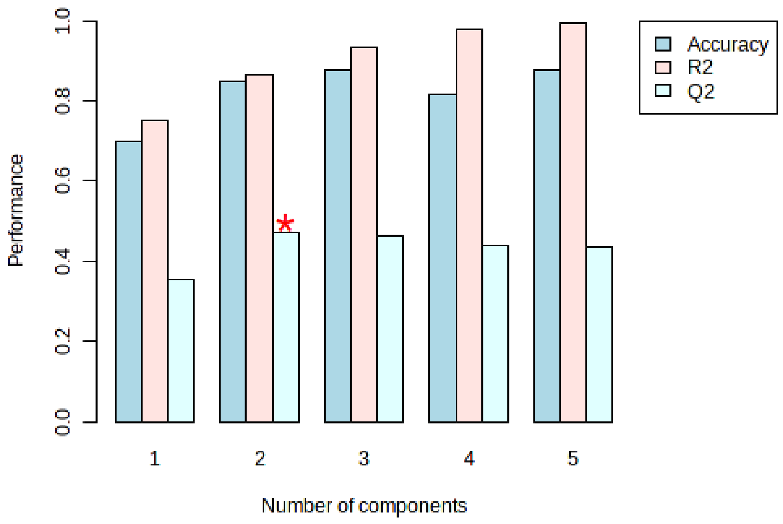

Figure S10. Cross validation of PLS-DA model. The values of R2 (0.87) and Q2 (0.47) revealed satisfactory goodness of fit and goodness of prediction, respectively (* = best Q2 measured performance).

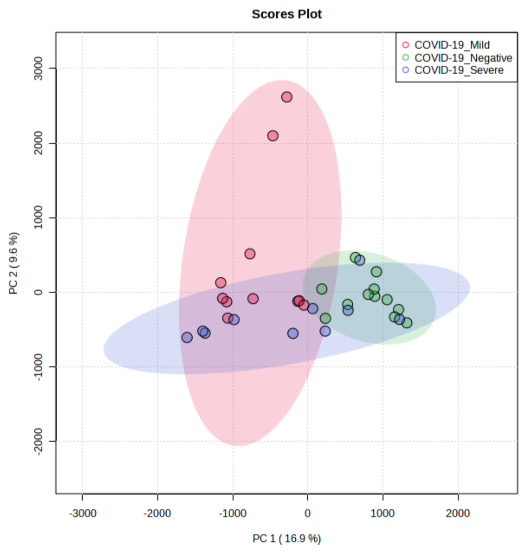

Figure S11. PCA model. PCS summarizing the most prominent peptides contributing to the observed phenotypic variations in the COVID-19 peptidome.

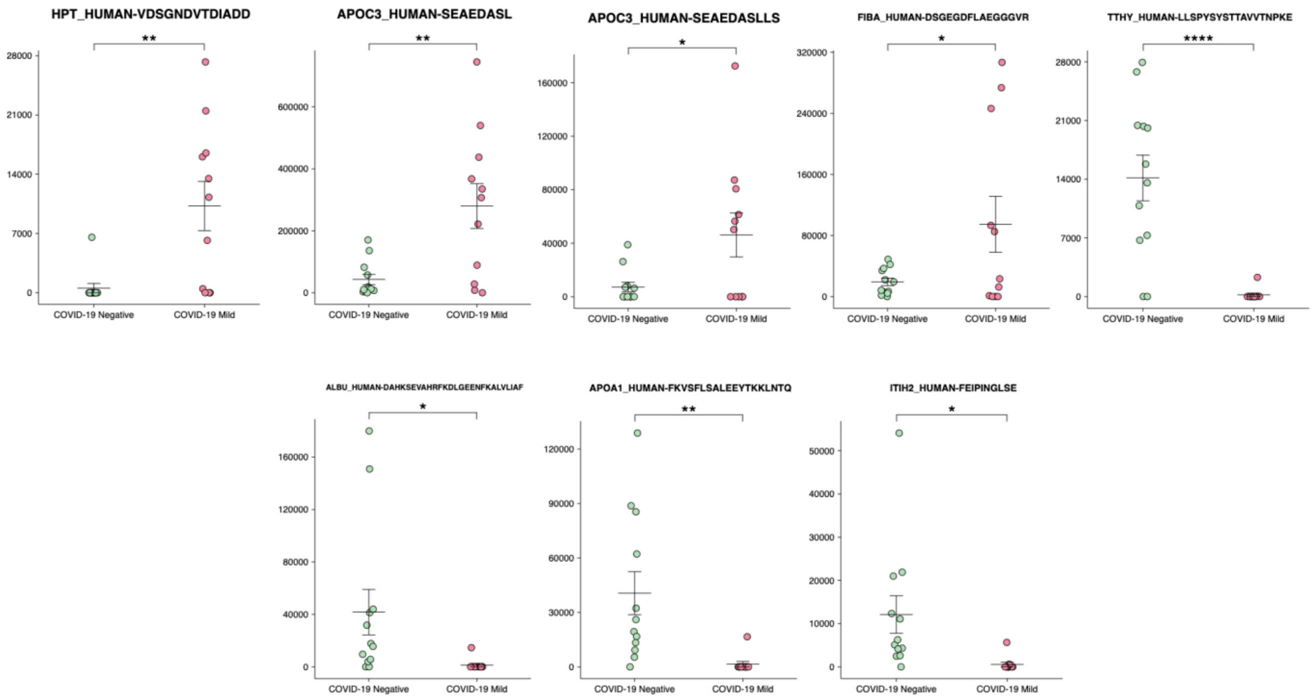

Figure S12. Boxplots of most modulated peptides in COVID-19 mild patients compared to negative controls (* p-value < 0.05; ** p-value < 0.01; **** p-value < 0.0001).

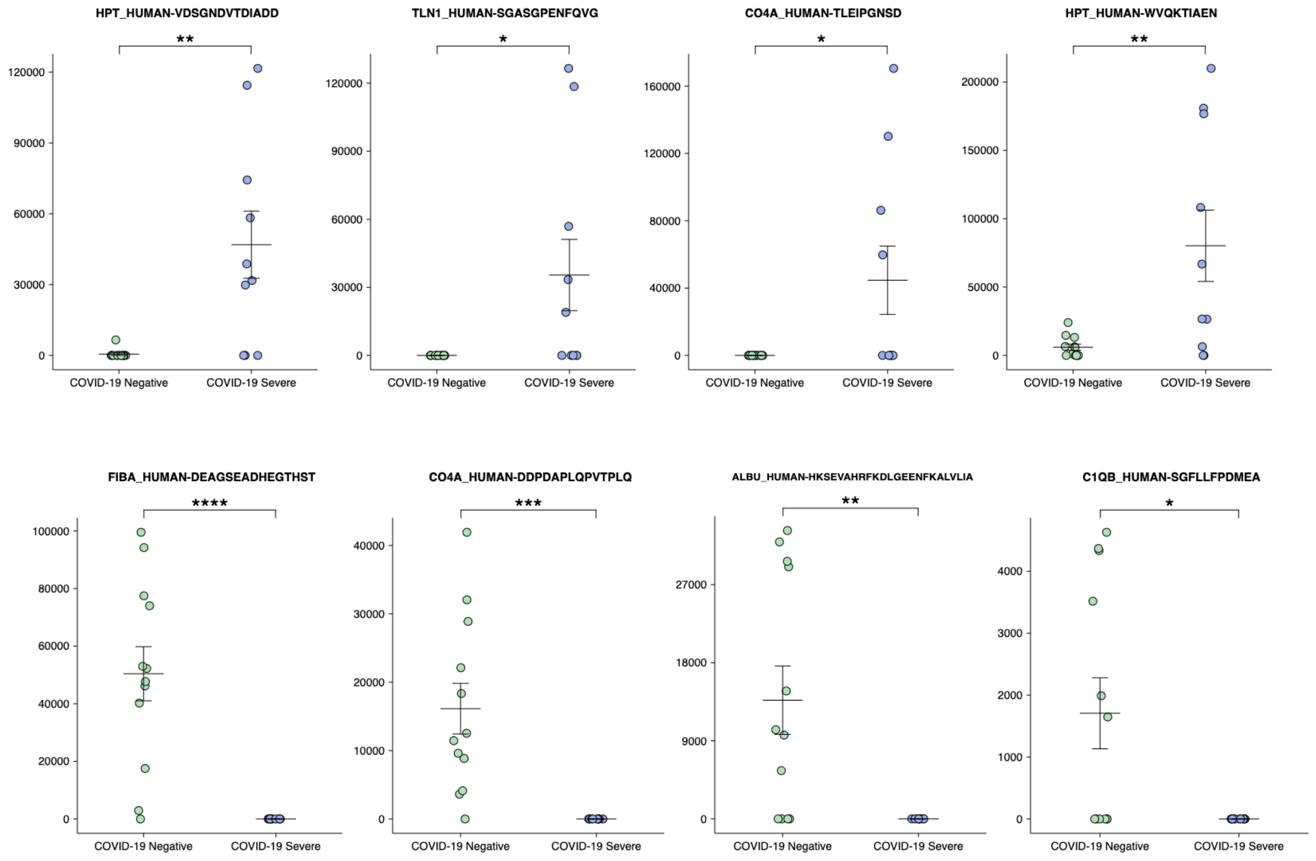

Figure S13. Boxplots of most modulated peptides in COVID-19 severe patients compared to negative controls (* p-value < 0.05; ** p-value < 0.01; *** p-value < 0.001; **** p-value < 0.0001).
